# Proteomic Identification of Protein Targets for 15-Deoxy-Δ^12,14^-Prostaglandin J_2_ in Neuronal Plasma Membrane

**DOI:** 10.1371/journal.pone.0017552

**Published:** 2011-03-18

**Authors:** Yasuhiro Yamamoto, Kenkichi Takase, Junji Kishino, Megumi Fujita, Noboru Okamura, Toshiyuki Sakaeda, Masafumi Fujimoto, Tatsurou Yagami

**Affiliations:** 1 Division of Physiology, Department of Pharmaceutical Health Care, Faculty of Pharmaceutical Sciences, Himeji Dokkyo University, Himeji, Japan; 2 Discovery Research Laboratories, Shionogi and Co., Ltd., Osaka, Japan; 3 Department of Clinical Pharmacy, School of Pharmaceutical Sciences, Mukogawa Women's University, Nishinomiya, Japan; 4 Center for Integrative Education of Pharmacy Frontier, Kyoto University Graduate School of Pharmaceutical Sciences, Kyoto, Japan; 5 Laboratory of Applied Pharmacology, Faculty of Pharmacy, Chiba Institute of Science, Choshi, Japan; University of Nebraska, United States of America

## Abstract

15-deoxy-Δ^12,14^-prostaglandin J_2_ (15d-PGJ_2_) is one of factors contributed to the neurotoxicity of amyloid β (Aβ), a causative protein of Alzheimer's disease. Type 2 receptor for prostaglandin D_2_ (DP2) and peroxysome-proliferator activated receptorγ (PPARγ) are identified as the membrane receptor and the nuclear receptor for 15d-PGJ_2_, respectively. Previously, we reported that the cytotoxicity of 15d-PGJ_2_ was independent of DP2 and PPARγ, and suggested that 15d-PGJ_2_ induced apoptosis through the novel specific binding sites of 15d-PGJ_2_ different from DP2 and PPARγ. To relate the cytotoxicity of 15d-PGJ_2_ to amyloidoses, we performed binding assay [^3^H]15d-PGJ_2_ and specified targets for 15d-PGJ_2_ associated with cytotoxicity. In the various cell lines, there was a close correlation between the susceptibilities to 15d-PGJ_2_ and fibrillar Aβ. Specific binding sites of [^3^H]15d-PGJ_2_ were detected in rat cortical neurons and human bronchial smooth muscle cells. When the binding assay was performed in subcellular fractions of neurons, the specific binding sites of [^3^H]15d-PGJ_2_ were detected in plasma membrane, nuclear and cytosol, but not in microsome. A proteomic approach was used to identify protein targets for 15d-PGJ_2_ in the plasma membrane. By using biotinylated 15d-PGJ_2_, eleven proteins were identified as biotin-positive spots and classified into three different functional proteins: glycolytic enzymes (Enolase2, pyruvate kinase M1 (PKM1) and glyceraldehyde 3-phosphate dehydrogenase (GAPDH)), molecular chaperones (heat shock protein 8 and T-complex protein 1 subunit α), cytoskeletal proteins (Actin β, F-actin-capping protein, Tubulin β and Internexin α). GAPDH, PKM1 and Tubulin β are Aβ-interacting proteins. Thus, the present study suggested that 15d-PGJ_2_ plays an important role in amyloidoses not only in the central nervous system but also in the peripheral tissues.

## Introduction

Eicosanoids are divided into two groups, according to their mechanism of action: the conventional eicosanoids, *e.g*., prostaglandin D_2_ (PGD_2_) and the cyclopentenone-type PGs, *e.g*., 15-deoxy-Δ^12,14^-PGJ_2_ (15d-PGJ_2_). PGD_2_ has been considered to be a pro-inflammatory mediator in inflammatory diseases such as Alzheimer's disease (AD) and Asthma. In AD, PGD_2_ formation increased in the frontal cortex of the patients when compared with those of the healthy subjects [1]. AD is characterized pathologically by cortical atrophy, neurodegeneration and deposits of amyloid protein in the various regions of brain such as cerebral cortex [2]. Amyloid β (Aβ) generated PGD_2_ from cortical neurons before inflammation [3]. However, the toxicity of PGD_2_ via its GTP-binding protein-coupled PGD_2_ receptors does not occur. First, the PGD_2_ receptor blocker did not inhibit PGD_2_-induced neuronal cell death [4]. Second, little mRNA of the PGD_2_ receptor is observed in the rat [5] and human [6] cerebral cortex. Third, few binding sites of [^3^H]PGD_2_ were detected in the plasma membranes from rat cortices [4]. Fourth, the extent of specific [^3^H]PGD_2_ in total biding is much lower (30–40%) than that of [^3^H]15d-PGJ_2_ (>80%), although binding sites of PGD_2_ have been reported in synaptosomes of rat [7] and human brains [6]. Fifth, the LD_50_ value (8.2 µM) of PGD_2_ is much higher than the affinity for PGD_2_ receptor (dissociation constant  = 14 nM) [5]. Finally, PGD_2_ required a latent time to exert toxicity. PGD_2_ was non-enzymatically metabolized to prostaglandin J_2_ (PGJ_2_), Δ^12^-PGJ_2_ and 15d-PGJ_2_ [4]. Among PGD_2_ metabolites, 15d-PGJ_2_ exhibited most potent inflammatory effects [4]. Taken together, PGD_2_ appeared to mediate inflammation via 15d-PGJ_2_ in the amyloidoses.

The surface receptors specific for 15d-PGJ_2_ have not been identified, and 15d-PGJ_2_ is believed to be actively transported into cells. It possesses an α, β-unsaturated carbonyl group in the cyclopentane ring that can form covalent adducts with free thiols in proteins by Michael addition. 15d-PGJ_2_ covalently binds to Cys^285^ of its nuclear receptor [8], peroxysome-proliferator activated receptorγ (PPARγ) [9], [10]. Recently, 15d-PGJ_2_ has been implicated in the antiproliferation independently from PPARγ[11]. Moreover, 15d-PGJ_2_ inhibits the NF-κB–dependent gene expression through the covalent modification at Cys^179^ in IκB kinase [12]. Previously, we have found the novel binding sites of 15d-PGJ_2_ on the cell surface [4]. [^3^H]15d-PGJ_2_ bound specifically to plasma membranes of cortical neurons. Among PGD_2_ metabolites, 15d-PGJ_2_ exhibited the highest affinity for the specific binding sites. Other eicosanoids and PPAR agonists did not affect the specific binding sites. 15d-PGJ_2_ regulated cell numbers in primary cultures of rat cortical neurons. The neurotoxicity of 15d-PGJ_2_ was the most potent among PGD_2_ and its metabolites, whereas little effect of other eicosanoids and PPAR agonists was detected. In peripheral tissues, 15d-PGJ_2_ also exhibited toxicity independently of PPARγ. In response to basic fibroblast growth factor, bronchial smooth muscle cells (BSMC) proliferate and remodel airway in asthma [13]. 15d-PGJ_2_ inhibits proliferation in a PPARγ-independent manner[14]. Thus, the identification of cell surface targets for 15dPGJ_2_ is required to clear how 15d-PGJ_2_ induces cell toxicity and involves in amyloidoses.

In the present study, we identified cell surface targets for 15d-PGJ_2_ in cortical neurons. In general, glycolytic enzymes, molecular chaperones and cytoskeletone identified as membrane targets for 15d-PGJ_2_ are known to localize in the cytosol, but their roles on the cell surface have not been elucidated sufficiently. Here, we propose hypothetical role of membrane targets for 15d-PGJ_2_ on the cell toxicity and amyloidoses.

## Materials and Methods

### Materials

Dulbecco's modified Eagle's medium, Leibovitz's L-15 medium, Roswell Park Memorial Institute 1640 medium, MCDB, CS-C, trypsin, deoxyribonuclease I, fetal bovine serum (FBS), horse serum (HS), penicillin, and streptomycin were obtained from Invitrogen (Carlsbad, CA). Aβ (25–35) was purchased from Bachem AG (Bubendorf, Switzerland). [^3^H]PGD_2_ (115 Ci/mmol) and human hepatocytes was purchased from Perkin Elmer Life Science Products (Boston, MA). Human BSMC and human dermal fibroblasts were purchased from Lonza (Basel, Switzerland). PGD_2_, PGJ_2_, Δ^12^-PGJ_2_, 15d-PGJ_2_ and biotinylated 15d-PGJ_2_ were obtained from Cayman Chemicals (Ann Arbor, MI; Cabru, Milan, Italy). Immobiline™ DryStrip Gels (pH 3–10), Amersham ECL Plus™ Western Blotting Detection Reagents, were obtained from GE Healthcare Bio-Sciences Corp. (Piscataway, NJ). Iodoacetamide, dithiothreitol (DTT), ethyleneglycol bis tetraacetic acid (EGTA) and ATP (disodium salt) were from Sigma-Aldrich (Milan, Italy). Sequence grade modified trypsin was purchased from Promega (Madison, WI; Milan, Italy), and *N*-(1-pyrenyl) iodoacetamide was from Molecular Probes (Eugene, OR). Horseradish peroxidase-linked antibody against biotine was obtained from Cell Signaling Technology (Boston, MA). The protein concentration was measured using the BCA protein assay reagent obtained from Thermo Fisher Scientific. (Rockford, IL). All other chemicals were of reagent grade.

### Tissue cultures

All procedures were conducted in accordance with NIH guidelines concerning the Care and Use of Laboratory Animals and with the approval of the Animal Care Committee of the Himeji Dokkyo University. Rat cortical neurons, human BSMC, human hepatocytes and human dermal fibroblasts were cultured as previously reported [15]. Cerebral cortices from the cerebral cortex of day-19 Sprague-Dawley rat embryos were dissociated in isotonic buffer with 4 mg/ml trypsin and 0.4 mg/ml deoxyribonuclease I. Cells were plated at a density of 2.5×10^5^ cells/cm^2^ on poly-L-lysine-coated dishes in conditioning medium, Leibovitz's L-15 medium supplemented with 5% FBS and 5% horse serum at 37°C in 5% CO_2_ and 9% O_2_. On day 1 after plating, cultures were treated with 0.1 µM arabinosylcytosine C. On day 4, cortical cultures were immunostained with anti-MAP2 specific for neurons, anti-GFAP for astrocytes, and anti-microglial antigen (OX-42). Cultures prepared by this method, consisted of approximately 95% neurons. Human BSMC were cultured at a density of 3.5×10^3^ cells/cm^2^ on 48-well plates in Molecular, Developmental, and Cellular Biology medium supplemented with 5% FBS, 50 µg/ml gentamicin, 50 ng/ml amphotericin. Human hepatocytes were cultured at a density of 5×10^4^ cells/cm^2^ on 48-well plates in CS-C medium (Applied Cell Biology Research Institute) supplemented with 10% FBS. Human dermal fibroblasts were cultured at a density of 5×10^4^ cells/cm^2^ on 48-well plates in DMEM supplemented with 10% FBS, 50 units/ml penicillin, and 50 µg/ml streptomycin.

### Aggregation assessment of fAβ

A stock solution of fibrillar Aβ (25–35) (fAβ) was prepared by dissolving Aβ at 1 mM in deionized water and incubating Aβ at 37°C for 2–5 days to aggregate the peptide and stored at −20°C until use [16]. The aggregation state of fAβ was assessed in two ways. First, light microscopy was used to identify the presence of precipitated peptides both in stock solutions and after their addition to tissue culture wells; the observations were confirmed by three observers. Second, the aggregation state of fAβ was assessed by migration patterns after sodium dodecyl sulfate-polyacrylamide gel electrophoresis (SDS-PAGE). Samples of fAβ stock solutions were added to reducing buffer, heated at 100°C for 3 min, and electrophoresed on 15% SDS–PAGE at 70 V.

### Cell viability

Two different methods were employed for assessment of cell viability as previously reported [15]. First, the 3-(4,5-dimethylthiazol-2-yl)-2,5-diphenyl tetrazolium bromide dye (MTT) reduction assay reflecting mitochondrial succinate dehydrogenase activity was employed. Second, residual cells were counted according to morphologic criteria; neurons with intact neurites and a smooth, round soma were considered viable, whereas those with degenerated neurites and an irregular soma were considered nonviable. BSMC with extended cell bodies and their bright phase-contrast appearance were considered viable, whereas those with shrank and round cell bodies were considered nonviable.

### Cell fractionation

Cell fractionation was performed as previously reported [17]. Cerebral cortices from rat brains were homogenized in 3 volumes of ice-cold STEA solution (0.25 M sucrose, 5 mM Tris-HCl (pH 7.5), 1 mM EGTA and 50 karikllein units/ml aprotinin). The homogenate was filtered through three meshes and centrifuged at 700×g for 10 min. Fractionations of nuclear and plasma membrane; The pellet was resuspended in 120 ml of STEA solution by gentle homogenization, and the resuspension was dispersed in 1080 ml of isosmotic Percoll solution (15.7% Percoll, 0.25 M sucrose, 1 mM EGTA, 50 karikllein units/ml aprotinin and 10 mM Tris HCI (pH 7.5)). The mixture was centrifuged at 35,000×g for 30 min. The resulting pellet was suspended in HEA solution (50 mM Hepes-NaOH (pH 7.4), 1 mM EGTA and 50 kIU/ml aprotinin) as the nuclear fraction. On the other hand, the second band from the surface in the supernatant was collected, washed by dilution with 2–3 volumes of HEA solution and centrifuged at 10,000×g for 30 min. The pellet was suspended in HEA solution as the plasma membrane fraction and stored in liquid nitrogen until used [18]. Fractionations of cytosol and microsome: The supernatant was centrifuged at 7,000×g for 10 min. The resulting supernatant was recentrifuged at 100,000×g for 1 h. The pellet was used as the microsomal fraction. The supernatant was used as the cytosolic fraction.

### Binding assay of [^3^H]15d-PGJ_2_

Binding assay of [^3^H]15d-PGJ_2_ were performed as previously reported [18]. The standard reaction mixture of 10 nM [^3^H]15d-PGJ_2_ contained 50 mM Tris-HCl buffer (pH 8.0), 100 mM NaCl and plasma membranes (10 µg) in a total volume of 100 µl. Incubation was initiated by addition of the reaction mixture to plasma membranes, and was carried out at 4°C for 24 h. We determined non-specific binding by performing incubations with [^3^H]15d-PGJ_2_ in the presence of 100 µM unlabeled 15d-PGJ_2_. The specific binding was calculated by subtraction of the non-specific binding from the total binding. Data are expressed as means ± standard error of the mean (S.E.M.) values (n = 4).

### Protein separation by two-dimensional electrophoresis

Membrane preparation and binding assay of biotinylated 15d-PGJ_2_ were conformed to “**Binding assay of [^3^H]15d-PGJ_2_**”. The standard reaction mixture of 1 µM biotinylated 15d-PGJ_2_ contained 50 mM Tris-HCl buffer (pH 8.0), 100 mM NaCl and plasma membranes (400 µg) in a total volume of 4 ml. Incubation was initiated by addition of the reaction mixture to plasma membranes, and was carried out at 4°C for 24 h in the presence or absence of unlabeled 15d-PGJ_2_. We determined non-specific binding by performing incubations with biotinylated 15d-PGJ_2_ in the presence of 100 µM unlabeled 15d-PGJ_2_. According to the method of Toda and Kimura [19], two-dimensional electrophoresis was performed with the CoolPhoreStar Horizontal Gel Electrophoresis Unit IPG-IEF (Anatech: Tokyo, JP). The samples containing 400 µg of membrane lysates were dissolved in a rehydration buffer (5 M urea, 2 M thiourea, 2%(w/v) CHAPS, 2%(w/v) SB3-10, 2% Pharmalytes and 65 mM DTT) for the first dimensional isoelectric focusing (IEF). The pH range of the IEF was 3–10. Before IEF was performed, the gel strips were incubated with a swelling buffer (6 M urea, 2 M thiourea, 2%(w/v) TritonX-100, 2%(w/v) SB3-10, 2% Pharmalytes, 2.5 mM acetic acid, 0.0025% BPB and 13 mM DTT). After IEF was performed, the gel strips were incubated with an SDS buffer (6 M urea, 32 mM DTT, 2%(w/v) SDS, 0.0025% BPB, 30%(v/v) glycerol, and 25 mM Tris-HCl pH 6.8) for 10 min, and then with an alkylation buffer (6 M urea, 243 mM iodoacetamide, 2%(w/v) SDS, 0.0025% BPB, 30%(v/v) glycerol, and 25 mM Tris-HCl pH 6.8) for 10 min. For the second dimensional electrophoresis, polyacrylamide gel (12% acrylamide, 0.4% bis-acrylamide, 10.6% glycerol, 0.1% SDS, 1.2% APS, 0.1% (v/v) TEMED and 369 mM Tris-HCl pH 8.8) was used. All procedures followed the manufacturer's protocol. Separated proteins were then fixed in the gel using 1) 40% methanol and 10% acetic acid, 2) 10% methanol and 7% acetic acid, and 3) 10% methanol and 8% acetic acid. Then, they were stained with SYPRO Ruby protein gel stain, and scanned using the FluoroPhoreStar® 3000 (Anatech: Tokyo, JP). The protein spots were visualized by Progenesis Same Spots (Nonliner Dynamics Ltd: Newcastle upon Tyne, UK). For immunoblotting, gels were transferred to polyvinylidene fluoride membranes (Millipore Co., Bedford, USA). The membranes were incubated with phosphate-buffered saline containing 0.1% Tween20 (PBS/Tween) and 5% skim milk for blocking and washed with PBS/Tween. This procedure was followed by the addition of horseradish peroxidase-conjugated anti-biotin antibody and ECL reagents (GE Healthcare Bio-Sciences). The spots were visualized by LAS-3000 (Aisin Seiki Co., Ltd., Aichi, Japan).

### Identification of 15d-PGJ_2_-targeted proteins

Gel pieces were washed in 50 mM ammonium bicarbonic acid containing 50% acetonitrile for 10 min, twice. Then, they were dried in block incubator Bl-516S (ASTEC Co., ltd.; Tokyo, JP) at 95°C for 10 min. Each sample was proteolyzed with 10 µl 1 mM ammonium bicarbonic acid containing 200 ng trypsin overnight at 37°C. The peptide in each gel was extracted with 50% acetonitrile containing 0.1% TFA followed by sonication for 15 min. The supernatant was collected, and peptides were further extracted with 75% acetonitrile containing 0.1% TFA followed by sonication for 15 min. Peptide extracts were concentrated to <10 µl using Speedvac concentrator. Then, they were desalted with Ziptip (Millipore Co.) and mixed with an equal volume of 5 mg α-cyano-4hydroxycinnamic acid (Shimadzu GLC ltd.; Tokyo, JP) dissolved in 0.5 ml 50% acetonitrile containing 0.1% TFA. One micro liter samples were spotted onto a matrix assisted laser desorption/ionization (MALDI) plate. After air drying, spots were identified by MALDI time of flight mass spectrometry (MALDI-TOF MS: Shimazu, AXIMA TOF^2TM^). MS spectra were collected over m/z 500–3500. The acquisition parameters were Tunig mode: Reflectron, Mass range: 1–3500, Max Laser Rep Rate: 10.0, CID: off, Power: 75, Profiles: 200, Shots: 5, Ion gate: Blank 900, P. Ext: 2500, Scenario: Advanced, Profile average: All profiles, Peak width: 2 chans, Smoothing method: Gaussian, Smoothing filter width: 2 chans, Baseline filter width: 16 chans, Peak detection method: Thresh hold Apex, Thresh hold offset 0.500 mV, Use monoisotopic peak picking, Minimum mass 500, Maximam mass: 3500, Resolution of the MS analyzer was 1,000 (0–1k Da), 5,000 (1 kDa–2 kDa) and 10,000 (>2 kDa).Minimum isotope: 1, Maximum intensity variation: 90 and Overlapping distributions Minimum peak percent: 10. Proteins were identified with the MASCOT (Matrix Science, London) searching algorithms using the Swiss-plot database. Probability-based MOWSE scores were estimated by comparison of search results against estimated random match population and were reported as-10* log 10(*p*), where *p* is the absolute probability. Scores greater than 50 were considered significant, meaning that for scores higher than 50 the probability that the match is a random event is lower than 0.05. The sequence version of the Swiss-Prot were heat shock cognate 71 kDa protein (Hspa8): 1, Internexin α: 2, Tubulin β2b: 1, glial fibrillary acidic protein (GFAP): 2, keratin, type I cytoskeletal 20 (CK20): 2, T-complex protein 1 subunit α (TCP1α): 1, pyruvate kinase M1 (PKM1): 3, Enolase 1: 4, Enolase 2: 2, Actin β: 1, F-actin-capping protein subunit α-2 (CapZα2): 1 and glyceraldehyde-3-phosphate dehydrogenase (GAPDH): 3. The interrogation parameters were Type of search: Peptide Mass Fingerprint, Enzyme: Trypsin, Fixed modifications: Carbamidomethyl (C), Variable modifications: Gln->pyro-Glu (N-term Q), Glu->pyro-Glu (N-term E), Oxidation (M), Mass values: Monoisotopic, Protein Mass: Unrestricted, Peptide Mass Tolerance: ±0.5 Da, Peptide Charge State: 1+ and Max Missed Cleavages: 1. Angiotensin II and ACTH were used as an internal standard. All protein identifications were in the expected size and PI range based on position in the gel.

### Western blotting

The standard reaction mixture of 1 µM biotinylated 15d-PGJ_2_ contained 50 mM Tris-HCl buffer (pH 8.0), 100 mM NaCl and plasma membranes (400 µg) in a total volume of 4 ml. Incubation was initiated by addition of the reaction mixture to plasma membranes, and was carried out at 4°C for 24 h. Membrane lysates were incubated with Streptavidin Agarose beads (Invitrogen, Carlsbad, CA) at room temperature for 30 min. The beads were rinsed three times with lysis buffer. The proteins were eluted by boiling the beads in Laemmli sample buffer and analysed by SDS-PAGE followed by immunodetection with antibodies to GAPDH (rabbit polyclonal, abcam [ab9485], Cambridge, UK), PKM1 (goat polyclonal, abcam [ab6191]), Enolase 2 (goat polyclonal, Santa Cruz [sc-31859], Santa Cruz, CA), Tubulin β (rabbit polyclonal, Santa Cruz [sc-9104]), TCP1α (mouse monoclonal, Enzo Life Sciences [ADI-CTA-191-D], New York, NY), Internexin α (mouse monoclonal, Millipore [MAB5224], Billerica, MA) and Actin β (mouse monoclonal, abcam [ab8226]). This procedure was followed by the addition of horseradish peroxidase-conjugated secondary antibody and ECL reagents.

### Statistical analysis

Data are given as means ± S.E.M. (n =  number of observations). Data were analyzed statistically by use of Student's non-paired *t* test for comparison with the control group, and data on various inhibitors and blocker groups were analyzed statistically by use of two-way ANOVA followed by Dunnett's test for comparison with the PG group (15d-PGJ_2_, Δ^12^-PGJ_2_, PGJ_2_, PGD_2_ and 15d-PGD_2_). The half maximal inhibitory concentration (IC_50_), the half maximal lethal dose (LD_50_) and the half maximal lethal time (LT_50_) were calculated by Microsoft Excel Fit.

## Results

### Susceptibilities of various cell lines to amyloid protein

**Figure 1:**
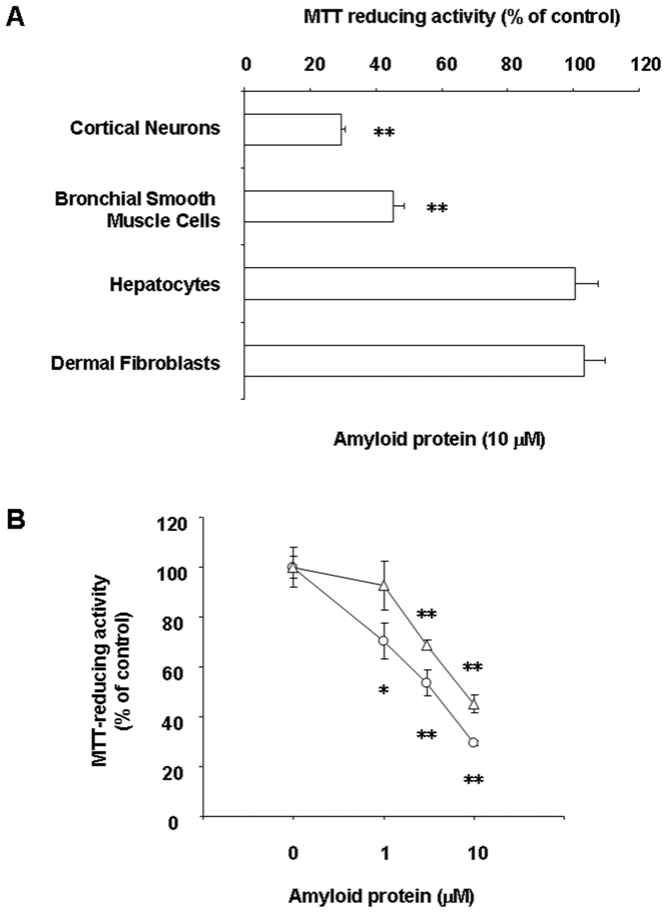
Effect of amyloid protein on the cell viability. (A) Cortical neurons, BSMC, hepatocytes and dermal fibroblasts were treated with 10 µM fAβ (25–35) viability. (B) Cortical neurons (circles) and BSMC (triangles) were treated with fAβ (25–35) or vehicle (ionized water) at the indicated concentrations. MTT reducing activity was determined 48 h later. MTT reducing activity was determined 48 h later. Data are expressed as means ± S.E.M. (n = 4). *P<0.05, **P<0.01, compared with control (vehicle) by ANOVA followed by Dunnett's test.

Sensitivities of various cell lines to amyloid protein were examined in the central nervous system and peripheral tissues. Cortical neurons, BSMC, hepatocytes and dermal fibroblasts were exposed to fAβ or vehicle (ionized water) for 48 h, and their viability was quantified by the MTT-reducing activity. In comparison with vehicle, fAβ significantly reduced the viability of cortical neurons and BSMC at 10 µM. On the other hand, fAβ did not significantly affect the viability of hepatocytes and dermal fibroblasts (Figure 1A). In neuronal cells and BSMC among tested cell lines, amyloid protein inhibited the cell viability in a concentration-dependent manner (Figure 1B).

### Sensitivities of various cell lines to 15d-PGJ_2_

**Figure 2:**
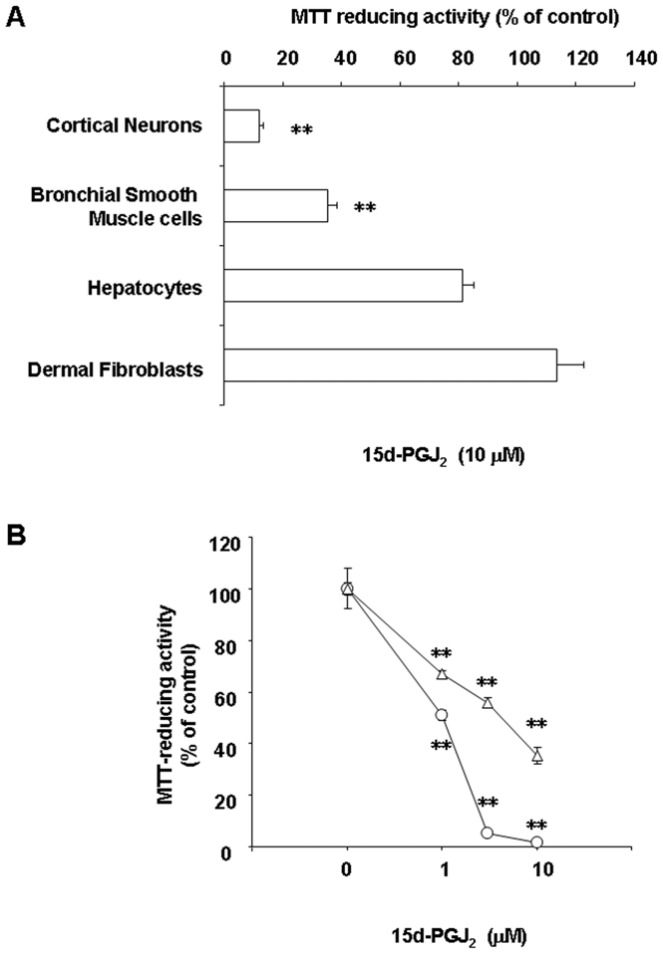
Effect of 15d-PGJ_2_ on the cell viability. (A) Cortical neurons, BSMC, hepatocytes and dermal fibroblasts were treated with 10 µM 15d-PGJ_2_ or vehicle (0.1% ethanol). (B) Cortical neurons (circles) and BSMC (triangles) were treated with 15d-PGJ_2_ at the indicated concentrations. MTT-reducing activities of cortical neurons and other cells were determined 24 h or 48 h later, respectively. Data are expressed as means ± S.E.M. (n = 4). *P<0.05, **P<0.01, compared with control by ANOVA followed by Dunnett's test.

We examined susceptibilities to 15d-PGJ_2_ in cortical neurons, BSMC, hepatocytes and dermal fibroblasts. These cell lines were exposed to 15d-PGJ_2_ or vehicle (0.1% ethanol), and their viability was quantified by the MTT-reducing activity. In comparison with vehicle, 15d-PGJ_2_ significantly reduced the viability of cortical neurons and BSMC at 10 µM. On the other hand, 15d-PGJ_2_ did not significantly affect cell viability of hepatocytes and dermal fibroblasts (Figure 2A). As well as amyloid protein, 15d-PGJ_2_ also reduced the cell viability of neuronal cells and BSMC, but neither hepatocytes nor dermal fibroblasts in a concentration-dependent manner (Figure 2B).

**Figure 3:**
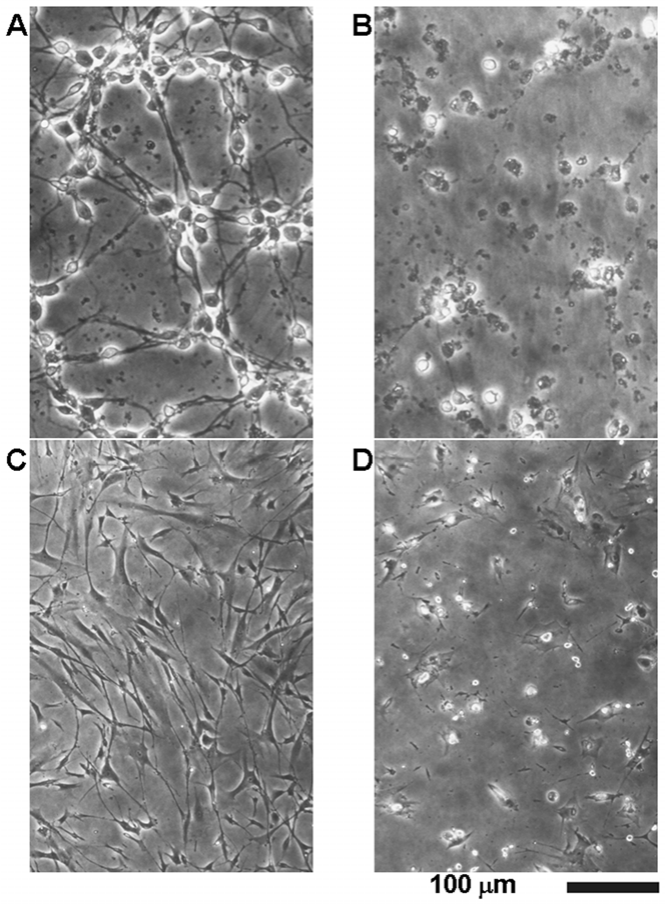
15d-PGJ_2_ induced morphorogical degeneration in cortical neurons and BSMC. Cortical neurons (A and B) and BSMC (C and D) were treated with vehicle (A and C) or 10 µM 15d-PGJ_2_ (B and D). Vehicle was 0.1% ethanol. Cortical neurons and BSMC were examined by phase-contrast microscopy 24 h and 48 h later, respectively.

In control cultures, neurons had extended neurites and smooth, round cell bodies (Figure 3A). On the other hand, some cell bodies shrank and lost their bright phase-contrast appearance in 15d-PGJ_2_-treated cultures. By 24 h, there were markedly fewer cells, and extensive debris was seen attached to the substratum (Figure 3B). In control cultures, BSMC extended cell bodies and exhibited their bright phase-contrast appearance (Figure 3C). When BSMC were cultured, we confirmed that the cell density was increased (data not shown). This increment was significantly prevented by 10 µM 15d-PGJ_2_ (Figure 3B and 3D). In 15d-PGJ_2_-treated cultures, some cell bodies shrank and became round (Figure 3D). Thus, there was a close correlation between susceptibilities to 15d-PGJ_2_ and amyloid protein, suggesting an involvement of 15d-PGJ_2_ in the amyloid protein-induced inflammation.

### Effects of PGD_2_ and Its metabolites on the viability of cortical neurons and BSMC

**Figure 4:**
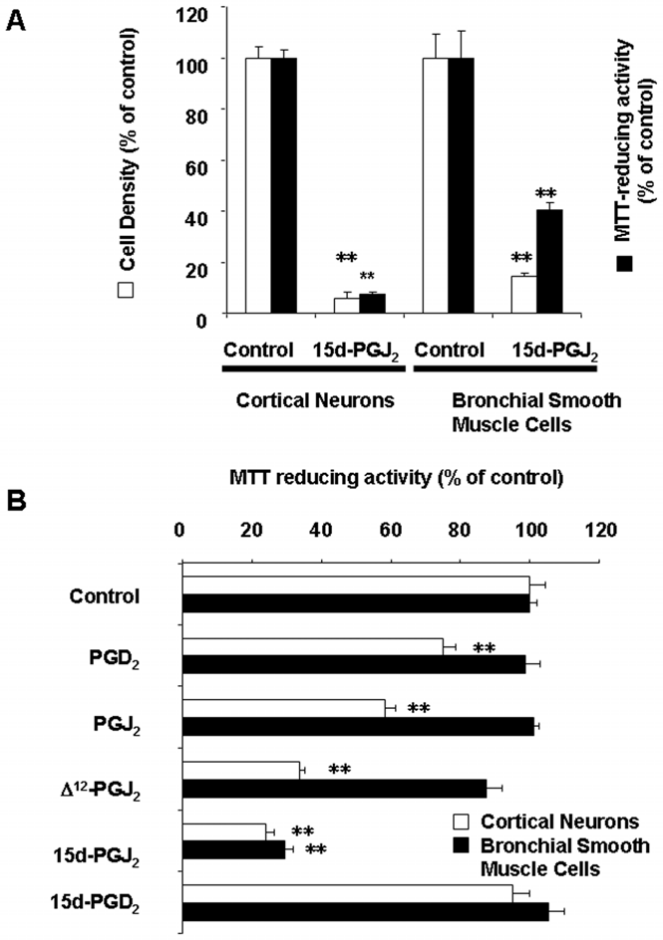
15d-PGJ_2_ downregulated cortical neurons and BSMC. (A) Cortical neurons and BSMC were treated with vehicle (control) or 10 µM 15d-PGJ_2_. Vehicle was 0.1% ethanol. Cell densities (open columns) and MTT-reducing activities (closed columns) in cortical neurons and BSMC were determined 24 h or 48 h later, respectively. (B) Cortical neurons and BSMC were treated with vehicle (control), PGD_2_, PGJ_2_, Δ^12^-PGJ_2_ or 15d-PGJ_2_ at 10 µM. Vehicle was 0.1% ethanol. MTT-reducing activities in cortical neurons (open columns) and BSMC (closed columns) were determined 8 h or 48 h later, respectively. Data are expressed as means ± S.E.M. (n = 4). **P<0.01, compared with control by ANOVA followed by Dunnett's test.

MTT assay is a colorimetric assay for measuring the activity of enzymes that reduce MTT or close dyes to formazan dyes. These reductions take place only when reductase enzymes in mitochondria are active, and therefore conversion is often used as a measure of viable (living) cells. Previously, we have reported that there was a linear relationship between cell density and MTT-reducing activities in cortical neurons [15]. As well as the MTT-reducing activity, the cell density was reduced by 10 µM 15d-PGJ_2_ in cortical neurons and BSMC (Figure 4A). MTT-reduction assay is also established for various cell types other than neurons to enable accurate, straightforward quantification of changes in their cell densities.

In most experiments, the neurotoxicity of 15d-PGJ_2_ was evaluated at 10 µM for 24 h in the presence of serum. Since PGD_2_ can be non-enzymatically metabolized to PGJ_2_, Δ^12^-PGJ_2_ and 15d-PGJ_2_ in the present culture medium [4], it is very difficult to compare their neurotoxic potencies. When serum was deprived from culture medium to decelerate the metabolism of PGD_2_, we have succeeded in detecting their neurotoxic hierarchy by the treatment with each PG at 10 µM for 8 h. We observed that serum-deprivation did not induced neuronal cell death within 8 h. The growth-inhibitory effect of PGD_2_ and its metabolites at 10 µM was 15d-PGJ_2_ > Δ^12^-PGJ_2_ > PGJ_2_ ≫ PGD_2_ in sequence (Figure 4B). On the other hand, 15-deoxy-Δ^12,14^-PGD_2_ (15d-PGD_2_) did not affect MTT-reducing activity of neuronal cells. In BSMC, 15d-PGJ_2_ significantly decreased MTT-reducing activities. Although Δ^12^-PGJ_2_ showed a tendency to decrease MTT-reducing activity, the inhibitory effect was significantly detected in neither 15d-PGD_2_, Δ^12^-PGJ_2_, PGJ_2_ nor PGD_2_.

### Specific binding sites of 15d-PGJ_2_ in the plasma membranes of cortical neurons and BSMC

**Figure 5:**
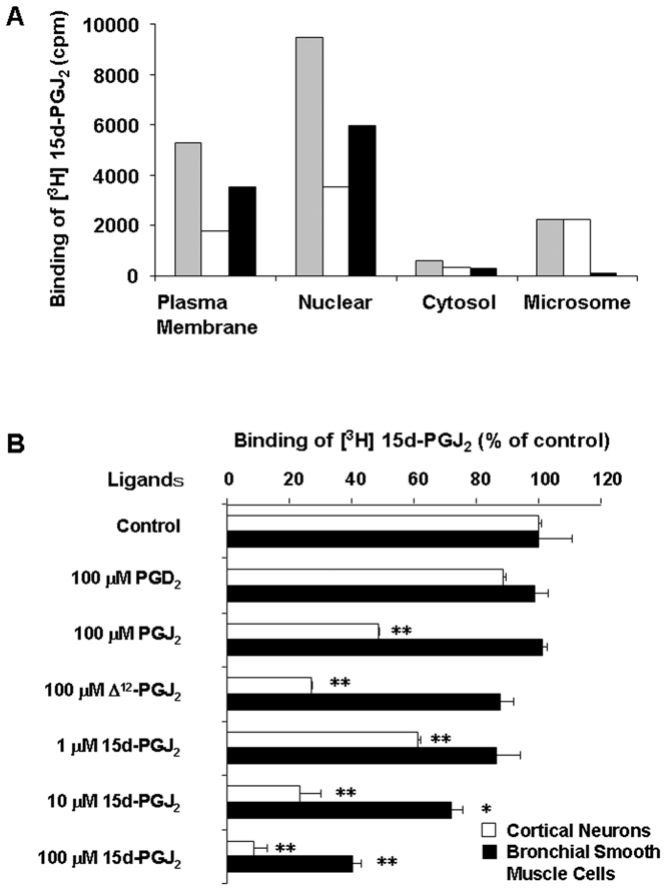
Binding assay of [^3^H]15d-PGJ_2_ to subcellular fractions. (A) Proteins fractionated to plasma membranes, nuclear, cytosol or microsome from cortical neurons were incubated with 10 nM [^3^H]15d-PGJ_2_ at 25°C for 1 h in the absence or presence of 10 µM unlabeled 15d-PGJ_2_. Total binding, nonspecific binding and specific binding were hatched columns, open columns and closed columns, respectively. Data are expressed as means (n = 2). (B) Effects of 15d-PGJ_2_ and its precursors on the binding of 10 nM [^3^H]15d-PG J_2_ to cortical neurons (open columns) and BSMC (closed columns). Plasma membranes (10 µg/protein) were incubated with [^3^H]15d-PGJ_2_ at 4°C for 24 h in the presence of unlabeled PGD_2_, PGJ_2_, Δ^12^-PGJ_2_ or 15d-PGJ_2_ at the indicated concentrations. The control value of [^3^H]15d-PGJ_2_ binding in cortical neurons and BSMC were 2523 cpm and 1309 cpm, respectively. Data are expressed as means ± S.E.M. (n = 4). *P<0.05, **P<0.01, compared with control by ANOVA followed by Dunnett's test.

Cortical neurons were fractionated into nuclear, plasma membrane, cytosol and microsome. Binding assay of [^3^H]15d-PGJ_2_ was performed at room temperature for 1 h. The ratio of specific binding of [^3^H]15d-PGJ_2_ to total binding were 78%, 66%, 45% and 4% in the fraction of plasma membrane, nuclear, cytosol and microsome, respectively (Figure 5A). Previously, we have reported the binding assay of [^3^H]15d-PGJ_2_ in the plasma membrane under optimal conditions at 4°C for 24 h [4]. The ratio of specific binding of [^3^H]15d-PGJ_2_ to total binding was more than 80% in the cortical neuron. The inhibitory effect of 15d-PGJ_2_-related compounds at 100 µM was 15d-PGJ_2_ > Δ^12^-PGJ_2_ > PGJ_2_ ≫ PGD_2_ in sequence (Figure 5B). 15d-PGJ_2_ displaced the specific binding of [^3^H]15d-PGJ_2_ in a concentration-dependent manner (Figure 5B). In BSMC, 15d-PGJ_2_ also inhibited the specific binding of [^3^H]15d-PGJ_2_ in a concentration-dependent manner (Figure 5B). The *IC_50_* value of 15d-PGJ_2_ to the specific binding of [^3^H]15d-PGJ_2_ in BSMC was 31 µM, and 20-fold higher than that (1.6 µM) in neuronal cells. The binding sites of 15d-PGJ_2_ in cortical neurons could also be recognized by Δ^12^-PGJ_2_ and PGJ_2_, whereas those in BSMC could be specifically done by 15d-PGJ_2_ (Figure 5B). In the two cells, the MTT-reducing activities of 15d-PGJ_2_ and its precursors were paralleled to the affinities of these ligands for the membrane specific binding sites of 15d-PGJ_2_.

### Comparison of the specific binding sites for [^3^H]15d-PGJ_2_ in plasma membranes to authentic receptors, DP1 and DP2

In peripheral tissues including nerves, chemoattractant receptor-homologous molecule expressed on Th2 cells has been identified as a type 2 receptor for PGD_2_ (DP2), and reported to be also a membrane receptor for 15d-PGJ_2_ [20]. We compared characterization of specific binding sites for [^3^H]15d-PGJ_2_ (SBJ) and DP2. According to LD_50_ and LT_50_, the apoptotic effect of 15d-PGJ_2_-related compounds was 15d-PGJ_2_ > Δ^12^-PGJ_2_ > PGJ_2_ > PGA_2_ ≫ PGD_2_ > 15d-PGD_2_ in sequence (Table 1). In the view of IC_50_, the affinity of 15d-PGJ_2_–related compounds for SBJ was 15d-PGJ_2_ > Δ^12^-PGJ_2_ > PGJ_2_ > PGA_2_ ≫ PGD_2_ > 15d-PGD_2_ in sequence (Table 1). On the other hand, the affinity of 15d-PGJ_2_–related compounds for DP2 was PGD_2_ > 15d-PGD_2_ >15d-PGJ_2_ > PGJ_2_ > Δ^12^-PGJ_2_ ≫ PGA_2_ in sequence (Table 1). In addition, the affinity of 15d-PGJ_2_–related compounds for DP1 was PGJ_2_ >PGD_2_ ≫ Δ^12^-PGJ_2_ >15d-PGJ_2_>15d-PGD_2_ > PGA_2_ in sequence (Table 1). Thus, the apoptotic effect of 15d-PGJ_2_-related compounds was correlated to their affinities for SBJ, but not to those for DP1 or DP2.

**Table 1:** Comparison of the specific binding sites for [^3^H]15d-PGJ_2_ in plasma membranes to authentic receptors, DP1 and DP2.

Ligand	Apoptosis	Apoptosis	SBJ	DP1	DP2
	LD_50_(µM)	LT_50_(h)	IC_50_(µM)	Ki (nM)	Ki (nM)
15d-PGJ_2_	1	4	1.6	280	3.2
Δ^12^-PGJ_2_	1	6	7	100	6.8
PGJ_2_	2	10	11	0.9	6.6
PGA_2_	5	>10	67	n.d.	23000
PGD_2_	>10	>10	>100	1.7	2.4
15d-PGD_2_	>10	>10	n.d.	6374	2.9

Apoptotic effects of 15d-PGJ_2_–related compounds were correlated to their affinities for DP1, DP2 and the specific binding sites for [^3^H]15d-PGJ_2_ (SBJ). LD_50_: The concentration of 15d-PGJ_2_–related compounds required to induce apoptosis in the half of neurons which were cultured for 24h in the absence of serum. LT_50_: The time of 10 µM 15d-PGJ_2_–related compounds required to induce apoptosis in the half of neurons which were cultured in the absence of serum. IC_50_: The concentration of 15d-PGJ_2_-related compounds required to inhibit half of the specific binding of [^3^H]15d-PGJ_2_ to SBJ. LD_50_, LT_50_ and IC_50_ were calculated from Yagami et al.[4]. These data on Ki: The Ki values of 15d-PGJ_2_-related compounds to DP1 and DP2 were referred from Sawyer et al[53].

### Isolation of Targets for 15d-PGJ_2_

**Figure 6:**
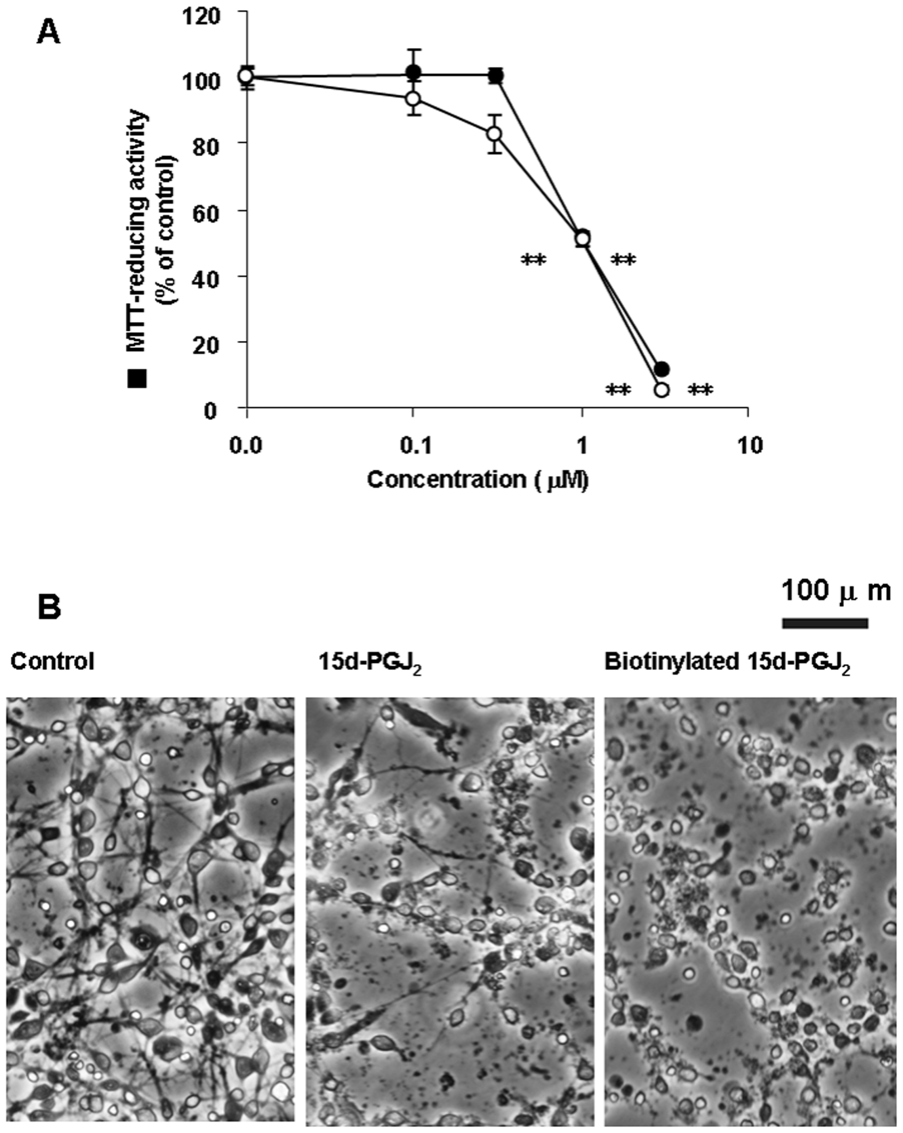
Biotinylated 15d-PGJ_2_ induced neuronal cell death. (A) Cortical neurons were treated with 15d-PGJ_2_ (open circles) or biotinylated 15d-PGJ_2_ (closed circles) at the indicated concentrations in the serum-free medium. MTT-reducing activities were determined 18 h later. Data are expressed as means ± S.E.M. (n = 4). **P<0.01, compared with control by ANOVA followed by Dunnett's test. (B) Cortical neurons were treated with vehicle (control), 3 µM 15d-PGJ_2_ or 3 µM biotinylated 15d-PGJ_2_ in the serum-free medium. Vehicle was 0.1% ethanol. Cortical neurons were photographed by phase-contrast microscopy 18 h later.

**Figure 7:**
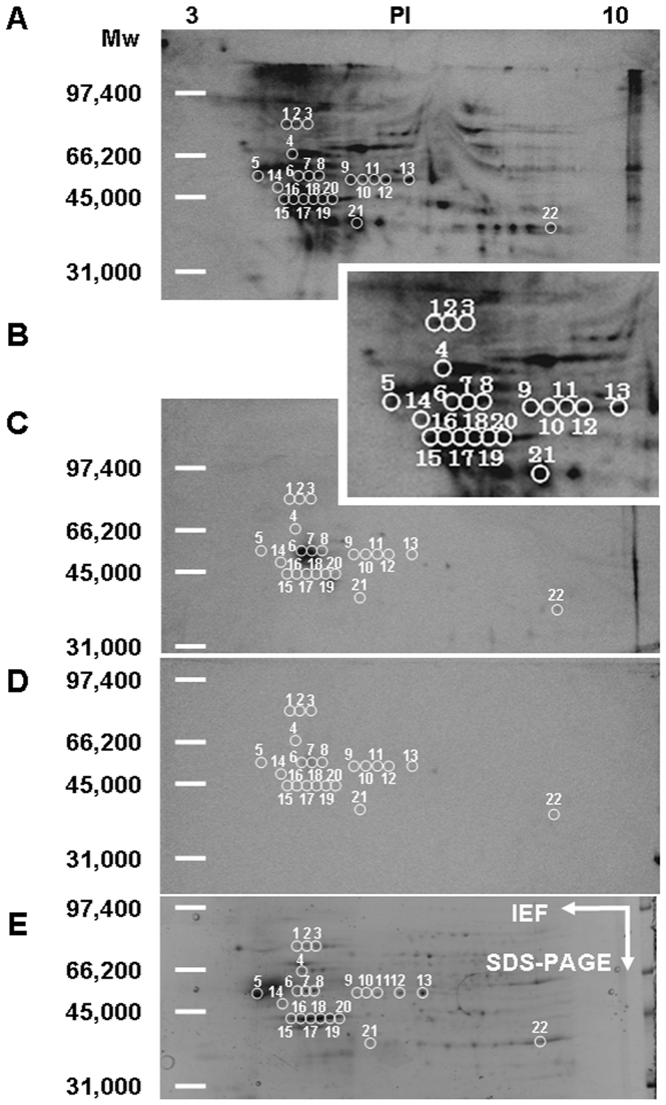
Identification of biotinylated 15d-PGJ_2_-modified proteins in the plasma membrane of cortical neurons. Western blotting: Membrane proteins (400 µg) were incubated with 1 µM biotinylated 15d-PGJ_2_ at 4°C for 24 h in the presence of vehicle (A), Magnified photograph including spot 1-21 (B), 10 µM unlabeled 15d-PGJ_2_ (C) and 100 µM unlabeled 15d-PGJ_2_ (D). SYPRO Ruby: Membrane proteins (400 µg) were incubated with 1 µM biotinylated 15d-PGJ_2_ at 4°C for 24 h in the presence of vehicle (E). The proteins were separated by isoelectrofocusing (pH 3-10) and then by SDS-PAGE. The white circles denote spots excised for subsequent identification by MALDI-TOF analysis, as described under Expreimental Procedures.

**Figure 8:**
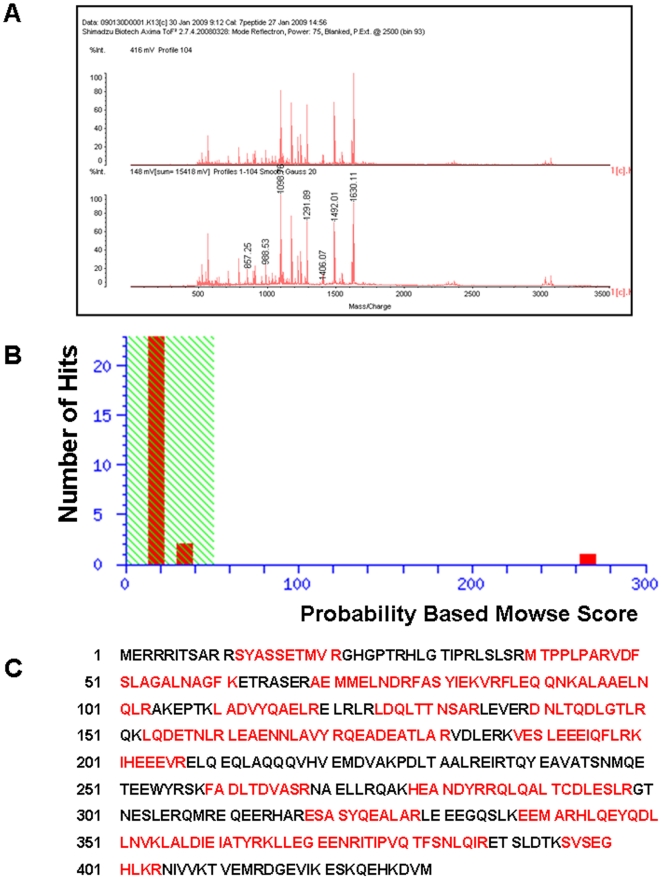
MALDI-TOF mass spectrum of the tryptic digest of spot 8. Spot 8 from Figure 7D was digested in gel with trypsin, and the resulting peptides were analyzed by MALDI-TOF MS as detailed in the experimental section. (A) Typical mass spectrum from a representative experiment. (B) Probability based Mowse Score. (C) Positions of matched peptides in the sequence of GFAP.

To identify target proteins for 15d-PGJ_2_, membrane proteins were labeled with biotinylated 15d-PGJ_2_ under the serum-free condition to reduce non-specific binding. Under this condition, biotinylated 15d-PGJ_2_ induced neuronal cell death in a concentration- dependent manner as well as 15d-PGJ_2_. Their LD_50_ values were almost 1 µM (Figure 6A). Biotinylated 15d-PGJ_2_ suppressed the extension of neurites and shrank cell bodies in a similar fashion to 15d-PGJ_2_ (Figure 6B). Next, neuronal plasma membranes were incubated with 1 µM biotinylated 15d-PGJ_2_ in the absence or presence of 15d-PGJ_2_ at the indicated concentrations. Then, membrane proteins modified with biotinylated 15d-PGJ_2_ were separated by two-dimensional gel electrophoresis. The patterns that were given by western blot analysis probed with anti-biotin antibody-HRP and SYPRO Ruby fluorescence staining are shown in Figure 7. Several biotinylated 15d-PGJ_2_-protein conjugates were detected as biotin-positive spots (Figure 7A and 7B). 15d-PGJ_2_ inhibited the modification of proteins with the biotinylated 15d-PGJ_2_ in a concentration-dependent manner (Figure 7C and 7D). At 100 µM, 15d-PGJ_2_ eliminated almost completely the biotin-positive spots (Figure 7D). After superimposition of both patterns, the SYPRO Ruby-stained proteins that coincided with the biotin-positive spots were excised from two-dimensional gels (Figure 7E), subjected to trypsin digestion, and then successfully analyzed by MALDI-TOF MS fingerprint analysis (Figure 8A).

### Identification of Targets for 15d-PGJ_2_

**Figure 9:**
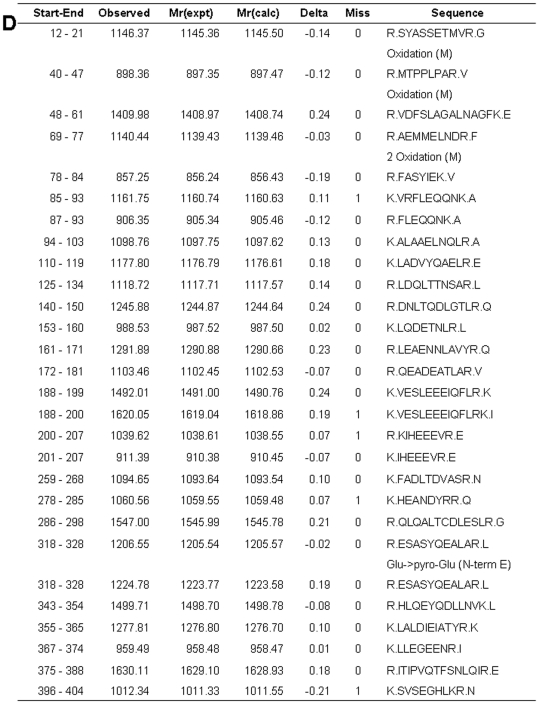
Peptide matches of spot 8 with GFAP. List of the monoisotopic masses of some of the peptides identified showing their position in the sequence of GFAP.

Spot 8 corresponding to a 50 kDa 15d-PGJ_2_-protein conjugate was one of the targets of the modification by biotinylated 15d-PGJ_2_, as seen in Figure 8. Using MASCOT, the probability based MOWSE score was 267 for GFAP (*p*<0.05) (Figure 8B), with 28 peptide matches (error ±0.02%) (Figure 9), which represents 56% sequence coverage (Figure 8C). Table 2 lists the identity of 22 protein spots, which could be identified in three independent experiments. The multiple gel spots for a single identification could be ascribed to posttranslational modification, such as phosphorylation. For example, spot 6 could contain 3 phosphorylation sites (T^129^, T^130^ and Y^283^), which represented the probability based MOWSE score59, 16 peptide matches, 32% sequence coverage. Spot 7 could contain 1 phosphorylation site (Y^283^), which represented the probability based MOWSE score 188, 31 peptide matches, 51% sequence coverage. On the other hand, the phosphorylation site of spot 8 was not detected. The identified proteins fall into several different functional classes, including glycolytic enzymes (Enolase 1, Enolase 2, GAPDH and PKM1), molecular chaperones (Hsp8a and TCP1α) and cytoskeltones (Tubulin β2b, Actin β, Internexin α, GFAP and CapZα2).

**Table 2:** Membrane proteins targeted for 15d-PGJ_2_.

No	Protein	Swiss-plot	MW	PI	Score	Matches	Coverage
1	Hspa8	P63018	71055	5.37	72	25/48	49
2	Hspa8	P63018	71055	5.37	100	13/20	23
3	Hspa8	P63018	71055	5.37	114	14/20	24
4	Internexin α	P23565	56224	5.20	89	11/20	18
5	Tubulin β2b	Q3KRE8	49931	4.78	99	11/20	21
6	GFAP	P47819	49984	5.35	97	14/40	40
7	GFAP	P47819	49984	5.35	292	31/40	51
8	GFAP	P47819	49984	5.35	267	28/39	56
9	CK20	P25030	49414	5.28	52	12/19	31
10	TCP1α	P28480	60835	5.86	51	10/33	22
11	PKM1	P11980	58331	6.63	52	9/29	26
12	Enolase1	P04764	47098	6.16	73	9/20	22
13	Enolase1	P04764	47098	6.16	62	8/20	23
14	Enolase 2	P07323	47111	5.03	50	7/20	19
15	Actin β	P60711	42052	5.29	73	7/20	52
16	Actin β	P60711	42052	5.29	65	9/37	28
17	Actin β	P60711	42052	5.29	79	8/19	23
18	Actin β	P60711	42052	5.29	58	9/38	27
19	Actin β	P60711	42052	5.29	48	8/34	23
20	Actin β	P60711	42052	5.29	82	9/24	26
21	CapZα2	Q3T1K5	33118	5.57	62	8/30	34
22	GAPDH	P04797	36090	8.14	73	10/36	27

Spots that were excised from the gel show in Figure 7E were identified by tryptic digestion and MALDI-TOF MS. Shown are the spot number, name of the identified protein, the accession number in the SwissProt database, the theoretical molecular mass and isoelectric point, the probability based MOWSE score, the number of peptides matched according to the Mascot database, the percentage of the protein sequence that is covered by the identified peptides.

**Figure 10:**
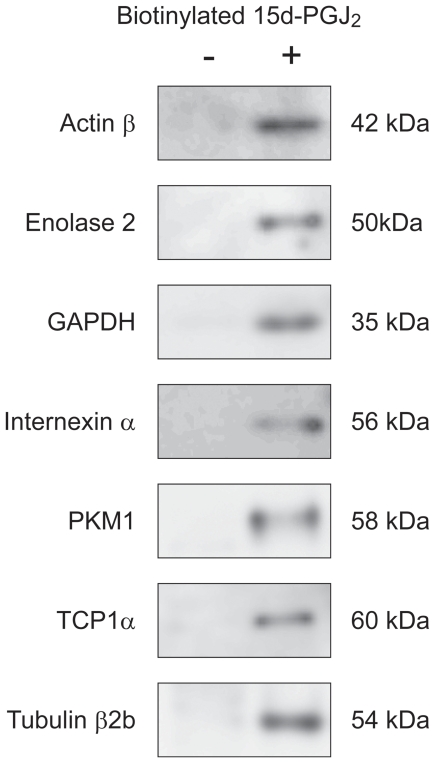
Interactions of 15d-PGJ_2_ with targets. Membrane proteins (400 µg) were incubated with 1 µM biotinylated 15d-PGJ_2_ at 4°C for 24 h. Membrane lysates were incubated with Streptavidin Agarose. The presence of targeted proteins was detected by immunoblot analysis, and the incorporation of biotinylated 15d-PGJ_2_ into immunoprecipitates was detected with ECL.

Next, we attempted to detect the 15d-PGJ_2_-target adducts in the plasma membranes exposed to the biotinylated 15d-PGJ_2_. by streptavidin agarose pull-down assays. Western blot revealed that 15d-PGJ_2_ interacted with Actin β, Enolase 2, GAPDH, Internexin α, PKM1, TCP1α and Tubulin β2b (Figure 10). Since plasma membranes were prepared from adult cerebral cortices including neurons and astrocytes, non-neuronal enolase1 and GFAP appeared to be derived from astrocytes.

### Regions homologous to the binding site of 15d-PGJ_2_ in targeted proteins

Several lines of evidences indicate the covalent binding sites of 15d-PGJ_2_ in previous target proteins. To ascertain whether the cysteine residue in the present target proteins responded to the covalent binding sites of 15d-PGJ_2_ in previous target proteins or not, homologous regions were searched (Table 3). As query sequences, we used the amino acid sequences of the previous target proteins, in which the covalent binding sites of 15d-PGJ_2_ are identified: Cys^374^ of Actin β (P60711) [21], Cys^269^ of c-Jun (NP_068607) [22], Cys^184^ of H-ras (NP_001091711) [23], Cys^179^ of IκB-kinase β (Q9QY78)^ 8^, Cys^285^ of PPARγ (NP_619725) [8], Cys^35^ and Cys^69^ of thioredoxin (NP_446252) [24]. Hspa8 contained Cys^603^ responded to the Cys^179^ of IκB-kinase β. The amino sequence of Hspa8 from Lys^597^ to Leu^610^ was homologous to that of IκB-kinase β from Lys^171^ to Leu^186^. Based on the comparison between the two sequences, the initial score, the optimal score and the identity were 15, 29 and 31%, respectively. In a similar fashion, Internexin α, Tubulin β2b, GFAP, CK20, TCP1α, PKM1, Enolase 1, Enolase 2, Actin β, CapZα2 and GAPDH contained the cysteine residue responded to Cys^69^ of thioredoxin, Cys^184^ of H-ras, Cys^269^ of c-Jun, Cys^69^ of thioredoxin, Cys^35^ of thioredoxin, Cys^35^ of thioredoxin, Cys^184^ of H-ras, Cys^184^ of H-ras, Cys^374^ of actin β, Cys^179^ of IκB-kinase β^8^, Cys^35^ of thioredoxin, respectively. Thus, the present target proteins also contained the cysteine residue responded to the previous covalent binding site of 15d-PGJ_2_, and exhibited homologous sequences around the specific binding site.

**Table 3:** Regions homologous to the binding site of 15d-PGJ_2_ in targeted proteins.

		Initial Score
Protein	Sequences	Optimal Score
		Identity
IκB-kinase β	Query 171 KELDQGSLCTSFVGTL 186	15
	| | | . . . | . . . . . . |	29
Hspa8	Sbjct 597 KELEK– –VCNPI I T KL 610	5/16 (31%)
Thioredoxin	Query 60 DDCQDVAADCE 70	16
	. . | . | | |	24
Internexin α	Sbjct 173 EEV QRLR ARCE 183	4/11 (36%)
H-ras	Query 183 SCKCV 187	14
	| | . | .	28
Tubulin β2b	Sbjct 126 SCDCL 130	3/5 (60%)
c-Jun	Query 261 RNRI AAS KCRKRKL 274	16
	| . . . . | . | . |	21
GFAP	Sbjct 284 RRQLQALTC DLE S L 297	4/14 (28%)
Thioredoxin	Query 59 VDDCQDVAADCEVK 72	8
	. . | | . | | . .	20
CK20	Sbjct 136 I KDAQ I ENARCVLQ 149	4/14 (28%)
Thioredoxin	Query 34 PCKMI KPFFH 43	18
	. | | . . . .	23
TCP1α	Sbjct 124 ACKEAVRYIN 133	2/10 (20%)
Thioredoxin	Query 35 CKMI 38	17
	| | . .	23
PKM1	Sbjct 165 CKVV 168	2/4 (50%)
H-ras	Query 178 GPGCMSCKCV L 188	17
	. . | . | | . | . |	37
Enolase 1	Sbjct 331 AAGE KSCNCL L 341	5/11 (45%)
H-ras	Query 183 SCKCVL 188	17
	. | . | . |	34
Enolase 2	Sbjct 336 ACNCLL 341	3/6 (50%)
Actin β	Query 356 WISKQEYDESGPSIVHRKCF 375	117
	| | | | | | | | | | | | | | | | | | | |	117
Actin β	Sbjct 356 WISKQEYDESGPSIVHRKCF 375	20/20 (100%)
IκB-kinase β	Query 169 YAKELDQGSL CT SFVGTLQ 187	21
	| . | | . . . . | | . . . .	38
CapZα2	Sbjct 131 YVKEHYPNGVCTVYGKKVD 149	5/19 (26%)
Thioredoxin	Query 35 CKMIKP 40	11
	| . . | |	28
GAPDH	Sbjct 245 CRLEKP 250	3/6 (50%)

Homologies were determined with Lipman-Pearson searching algorithms using the Swiss-plot database. As query sequences, we used the amino acid sequences of the previous target proteins, in which the covalent binding sites of 15d-PGJ_2_ are identified: Cys^374^ of Actin β (P60711) [50], Cys^269^ of c-Jun (NP_068607) [51], Cys^184^ of H-ras (NP_001091711) [20], Cys^179^ of IκB-kinase β (Q9QY78) [12], Cys^285^ of PPARγ (NP_619725) [8], Cys^35^ and Cys^69^ of thioredoxin (NP_446252) [52]. As subject sequences, we used the amino acid sequences of our target proteins. The listed sequences exhibited the highest score in the initial score, the optimal score and the identity.

## Discussion

Cortical neurons and BSMC sensitive to amyloid protein were susceptible to 15d-PGJ_2_. [^3^H]15d-PGJ_2_ bound specifically to the two cells, suggesting that 15d-PGJ_2_ played an important role in amyloidoses not only in the central nervous system but also in the peripheral tissues. The specific binding sites of [^3^H]15d-PGJ_2_ were detected in the neuronal subcellular fractions of nuclear, cytosol and plasma membrane, but not in the microsomal fraction. 15d-PGJ_2_ binds to the nuclear receptor, PPARγ [9] and the cytosolic protein, Ras [23]. In peripheral tissues including nerves, chemoattractant receptor-homologous molecule expressed on Th2 cells has been identified as a type 2 receptor for PGD_2_ (DP2), and reported to be also a membrane receptor for 15d-PGJ_2_ [20]. Contrary to its mRNA, little protein of DP2 has yet been detected in the central nerve. Furthermore, we ruled out the possibility that the specific binding site of 15d-PGJ_2_ in the plasma membrane of cortical neurons was DP2. First, few binding sites of [^3^H]PGD_2_ are detected in plasma membranes from rat cortices [4]. Although binding sites of [^3^H]?^12^-PGJ_2_ and [^3^H]PGJ_2_ are also detected in plasma membranes, those are displaced most potently by 15d-PGJ_2_ among PGD_2_ metabolites [4]. Second, a DP2 selective agonist, 15d-PGD_2_ do not affect the cell number of neuronal cells and BSMC (Figure 3B and Table 1). Third, the LD_50_ value (>10 µM) of PGD_2_ is much higher than the affinity for PGD_2_ receptor (dissociation constant  = 8.8 nM) [20].

**Figure 11:**
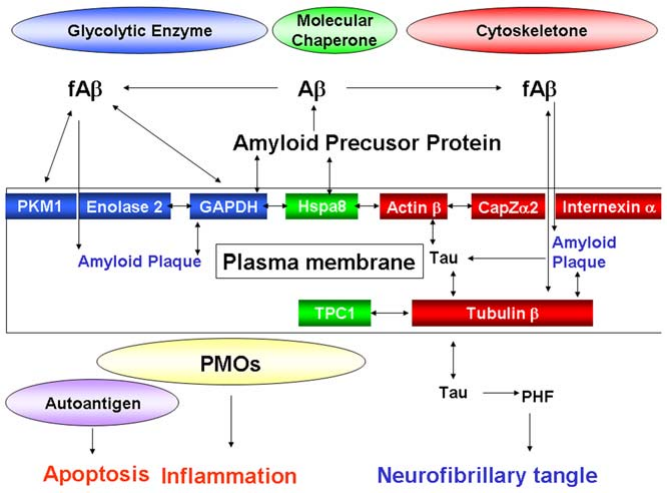
Hypothetical roles of targets for 15d-PGJ_2_ on amyloidoses. Membrane target proteins for 15d-PGJ_2_ were glycolytic enzymes (Enolase 2, PKM1 and GAPDH), molecular chaperones (Hsp8a and TCP1α), and cytoskeletal proteins (Actin β, CapZα2, Tubulin β and Internexin α). These proteins were factors associated with the two remarks of AD, the amyloid plaque and the neurofibrillary tangle. Beyond classical roles as glycolytic enzymes and molecular chaperones, GAPDH, Enolase2 and Hsp8a appear to form the complex of PMOs and contribute to the generation of reacting oxygen species by 15d-PGJ_2_.

In the present study, we identified membrane proteins targeted for 15d-PGJ_2_ including glycolytic enzymes, molecular chaperones and cytoskeletons (Table 2 and Figure 10). GAPDH, Enolase 1, Enolase 2 and PKM1 were previously believed to perform exclusively ‘house-keeping’ glycolysis. GAPDH is not only found in the cytoplasm, but also closely associated with the plasma membrane [25]. GAPDH catalyses the conversion of glyceraldehyde 3-phosphate to D-glycerate 1,3-bisphosphate. Reduction in glycolysis precedes cognitive dysfunction and is therefore believed to be an important early event in AD development [26]. Apart from its glycolytic role, overexpression of the particular membrane-associated GAPDH has a direct role in neuronal apoptosis [27] (Figure 11). GAPDH is located in amyloid plaques [28], interacts with the C-terminal region of amyloid precursor protein (APP) [29], and co-precipitates with fAβ[30]. Furthermore, GAPDH associates tightly with Enolase 2 and Hspa8, and makes up *trans*-plasma-membrane oxidoreductases (PMOs), the extracellular redox sensor for signaling external oxidative stress to the cell [31].

Enolase 1 and Enolase 2 belong to a superfamily of abundantly expressed carbon-oxygen lyases known for the catalysis of 2-phosphoglycerate to phosphoenolpyruvate. Ubiquitous enolase1 and neuron specific enolase 2 exist as monomers and also as dimmers on the neuronal membrane surface [32]. Recent studies have demonstrated that enolases possess different regulatory properties from glycolysis in the brain [33]. Enolase1 is one of the most consistently up-regulated and oxidatively modified proteins in brain of subjects of early-onset AD [34]. Enolase1 and enolase 2 are autoantigen targets in post-streptococcal autoimmune disease of central nervous system (Figure 11). The anti-enolase antibodies induce neuronal apoptosis [35]. Enolase 2 is part of neuronal PMOs, and the anti-enolase2 antibody can inhibit PMO activity on the plasma membrane [31].

Pyruvate kinase transfers a phophate from phosphoenolpyruvate to ADP. Pyruvate kinase is also defined as the autoantigen, and its antibodies induce neuronal apoptosis [35] (Figure 10). The significant increase in pyruvate kinase activity is found in frontal and temporal cortex of AD brains [36]. Pyruvate kinase is elevated in the cortical neurons undergoing Aβ-mediated apoptosis [37]. Pyruvate kinase is co-precipitated with fAβ [30]. Biotinylated 15d-PGJ_2_ binds to PKM1 in mesangial cells [38], supporting our results.

Hsp8a is dnaK-type molecular chaperone heat shock protein 72-ps1 in the PMO complex [31]. It is located in the cytoplasm [39], but nuclear localization and accumulation near or at the plasma membrane in stressed cells and in synaptosomal membranes has been observed [40]. Hsp8a binds to the cytoplasmic domain near the post-transmembrane region of APP (Figure 11).

TCP1α is a selective molecular chaperone in tubulin biogenesis, by that nascent tubulin subunits are bound to TCP1α and released in assembly competent forms. Cytoskeletal proteins are deficient and aggregated in AD. When TCP1α is related to its natural and specific substrate tubulin β, the ratio is significantly decreased in the temporal, frontal, parietal cortex and in thalamus of AD patients [41]. Relatively decreased molecular chaperoning of tubulin β by TCP1α is suggested to lead to misfolded tubulin aggregating and accumulating in plaques and tangles, a hallmark of AD (Figure 11).

Tubulin has been identified as a membrane component of synaptosomes and various plasma membranes. Both tubulin α and β have been shown to associate with the amyloid deposits of familial amyloidosis [42] and to bind to the Aβ sequence of APP [43]. Moreover, tubulin β is retained by a monomeric Aβ column [44], and co-precipitated with fAβ [30] (Figure 11). The tau protein interacts with tubulin to stabilize microtubules and promote tubulin assembly into microtubules. PGJ_2_ induces caspase-mediated cleavage of tau, generating Δtau, an aggregation prone form known to seed tau aggregation prior to neurofibrillary tangle formation [45]. Hyperphosphorylation of the tau protein (tau inclusions) can result in the self-assembly of tangles of paired helical filaments and straight filaments, which are involved in the pathogenesis of AD [46]. Biotinylated 15d-PGJ_2_ binds to tubulin β in mesangial cells [38], supporting our results.

AD-linked human Aβ synergistically enhances the ability of wild-type tau to promote alterations in the actin cytoskeleton (Figure 11) and neurodegeneration [47]. The ability of globular actin to rapidly assemble and disassemble into filaments is critical to many cell behaviors. F-actin-capping protein subunit α-2 (CapZα2) regulates growth of the actin filament by capping the barbed end of growing actin filaments (Figure 11). Members of the actin-depolymerizing factor (ADF)/cofilin family are important regulators of actin dynamics. ADF and cofilin's ability to increase actin filament dynamics is inhibited by their phosphorylation on Ser^3^ by LIM kinase 1 and other kinases [48] Aβ dystrophy requires LIM kinase 1-mediated phosphorylation of ADF/cofilin and the remodeling of the actin cytoskeleton [49]. Biotinylated 15d-PGJ_2_ covalently binds to actin β in various cells [38] other than neurons, supporting our results in neurons.

Internexin αis classified as a type IV neuronal intermediate filament. Internexin α also co-assembles with the neurofilament (NF) triplet proteins [50]. The protein is expressed by most, if not all, neurons as they commence differentiation and precedes the expression of the NF triplet proteins [51]. Although the interaction of internexin α with amyloid proteins has not yet been reported, Internexin α, and not NF triplet, ring-like reactive neurites are present in end-stage AD cases, indicating the relatively late involvement of neurons that selectively contain Internexin α (Figure 11). Another intermediate filament protein, GFAP is expressed exclusively in astrocytes. Aβ increased the total number of activated astrocytes, and elevated the expression of GFAP by Aβ-induced spontaneous calcium transients [52]. 15d-PGJ_2_ suppresses inflammatory response by inhibiting NF-κB signaling at multiple steps as well as by inhibiting the PI3K/Akt pathway independent of PPARγ in primary astrocytes [53].

In conclusion, membrane target proteins for 15d-PGJ_2_ were factors associated with the two remarks of AD, the amyloid plaque and the neurofibrillary tangle. Beyond classical roles as glycolytic enzymes and molecular chaperones, GAPDH, enolase 2 and Hsp8a can form the antioxidant complex of PMOs responded to the extracellular oxidative stress. 15d-PGJ_2_ might regulate the activity of PMOs during inflammation and degeneration. Apart from glycolysis, pyruvate kinase and enolase might be involved in the 15d-PGJ_2_–induced apoptosis as autoantigens. Thus, the present study sheds light on the ecto-enzymes targeted for 15d-PGJ_2_ as a prelude to the death receptor stimulated by 15d-PGJ_2_ or the antioxidant complex regulated by 15d-PGJ_2_.

## References

[1] Iwamoto N, Kobayashi K, Kosaka K (1989). The formation of prostaglandins in the postmortem cerebral cortex of Alzheimer-type dementia patients.. J Neurol.

[2] Selkoe DJ (1991). The molecular pathology of Alzheimer's disease.. Neuron.

[3] Yagami T, Ueda K, Asakura K, Sakaeda T, Kuroda T (2001). Effects of S-2474, a novel nonsteroidal anti-inflammatory drug, on amyloid beta protein-induced neuronal cell death.. Br J Pharmacol.

[4] Yagami T, Ueda K, Asakura K, Takasu N, Sakaeda T (2003). Novel binding sites of 15-deoxy-delta12,14-prostaglandin J2 in plasma membranes from primary rat cortical neurons.. Exp Cell Res.

[5] Wright DH, Nantel F, Metters KM, Ford-Hutchinson AW (1999). A novel biological role for prostaglandin D2 is suggested by distribution studies of the rat DP prostanoid receptor.. Eur J Pharmacol.

[6] Boie Y, Sawyer N, Slipetz DM, Metters KM, Abramovitz M (1995). Molecular cloning and characterization of the human prostanoid DP receptor.. J Biol Chem.

[7] Sasaguri T, Masuda J, Shimokado K, Yokota T, Kosaka C (1992). Prostaglandins A and J arrest the cell cycle of cultured vascular smooth muscle cells without suppression of c-myc expression.. Exp Cell Res.

[8] Shiraki T, Kamiya N, Shiki S, Kodama TS, Kakizuka A (2005). Alpha, beta-unsaturated ketone is a core moiety of natural ligands for covalent binding to peroxisome proliferator-activated receptor gamma.. J Biol Chem.

[9] Forman BM, Tontonoz P, Chen J, Brun RP, Spiegelman BM (1995). 15-Deoxy-delta 12, 14-prostaglandin J2 is a ligand for the adipocyte determination factor PPAR gamma.. Cell.

[10] Kliewer SA, Lenhard JM, Willson TM, Patel I, Morris DC (1995). A prostaglandin J2 metabolite binds peroxisome proliferator-activated receptor gamma and promotes adipocyte differentiation.. Cell.

[11] Ward JE, Gould H, Harris T, Bonacci JV, Stewart AG (2004). PPAR gamma ligands, 15-deoxy-delta12,14-prostaglandin J2 and rosiglitazone regulate human cultured airway smooth muscle proliferation through different mechanisms.. Br J Pharmacol.

[12] Rossi A, Kapahi P, Natoli G, Takahashi T, Chen Y (2000). Anti-inflammatory cyclopentenone prostaglandins are direct inhibitors of IkappaB kinase.. Nature.

[13] Gabazza EC, Taguchi O, Tamaki S, Takeya H, Kobayashi H (1999). Thrombin in the airways of asthmatic patients.. Lung.

[14] Patel HJ, Belvisi MG, Bishop-Bailey D, Yacoub MH, Mitchell JA (2003). Activation of peroxisome proliferator-activated receptors in human airway smooth muscle cells has a superior anti-inflammatory profile to corticosteroids: relevance for chronic obstructive pulmonary disease therapy.. J Immunol.

[15] Yagami T, Ueda K, Asakura K, Hata S, Kuroda T (2002). Human group IIA secretory phospholipase A2 induces neuronal cell death via apoptosis.. Mol Pharmacol.

[16] Yagami T, Takahara Y, Ishibashi C, Sakaguchi G, Itoh N (2004). Amyloid beta protein impairs motor function via thromboxane A2 in the rat striatum.. Neurobiol Dis.

[17] Yagami T, Tohkin M, Matsubara T (1990). Sex difference in adrenergic receptor-mediated glycogenolysis in rat livers.. Jpn J Pharmacol.

[18] Yagami T (1995). Differential coupling of glucagon and beta-adrenergic receptors with the small and large forms of the stimulatory G protein.. Mol Pharmacol.

[19] Toda T, Kimura N (1997). Standardization of protocol for immobiline 2-D PAGE and construction of 2-D PAGE protein database on World Wide Web home page.. Jpn J Electroph.

[20] Hata AN, Zent R, Breyer MD, Breyer RM (2003). Expression and molecular pharmacology of the mouse CRTH2 receptor.. J Pharmacol Exp Ther.

[21] Aldini G, Carini M, Vistoli G, Shibata T, Kusano Y (2007). Identification of actin as a 15-deoxy-delta12,14-prostaglandin J2 target in neuroblastoma cells: mass spectrometric, computational, and functional approaches to investigate the effect on cytoskeletal derangement.. Biochemistry.

[22] Perez-Sala D, Cernuda-Morollon E, Canada FJ (2003). Molecular basis for the direct inhibition of AP-1 DNA binding by 15-deoxy-delta 12,14-prostaglandin J2.. J Biol Chem.

[23] Oliva JL, Perez-Sala D, Castrillo A, Martinez N, Canada FJ (2003). The cyclopentenone 15-deoxy-delta 12,14-prostaglandin J2 binds to and activates H-Ras.. Proc Natl Acad Sci U S A.

[24] Shibata T, Yamada T, Ishii T, Kumazawa S, Nakamura H (2003). Thioredoxin as a molecular target of cyclopentenone prostaglandins.. J Biol Chem.

[25] Rogalski AA, Steck TL, Waseem A (1989). Association of glyceraldehyde-3-phosphate dehydrogenase with the plasma membrane of the intact human red blood cell.. J Biol Chem.

[26] Arias C, Montiel T, Quiroz-Baez R, Massieu L (2002). beta-Amyloid neurotoxicity is exacerbated during glycolysis inhibition and mitochondrial impairment in the rat hippocampus in vivo and in isolated nerve terminals: implications for Alzheimer's disease.. Exp Neurol.

[27] Ishitani R, Sunaga K, Hirano A, Saunders P, Katsube N (1996). Evidence that glyceraldehyde-3-phosphate dehydrogenase is involved in age-induced apoptosis in mature cerebellar neurons in culture.. J Neurochem.

[28] Tamaoka A, Endoh R, Shoji S, Takahashi H, Hirokawa K (1996). Antibodies to amyloid beta protein (A beta) crossreact with glyceraldehyde-3-phosphate dehydrogenase (GAPDH).. Neurobiol Aging.

[29] Schulze H, Schuler A, Stuber D, Dobeli H, Langen H (1993). Rat brain glyceraldehyde-3-phosphate dehydrogenase interacts with the recombinant cytoplasmic domain of Alzheimer's beta-amyloid precursor protein.. J Neurochem.

[30] Verdier Y, Huszar E, Penke B, Penke Z, Woffendin G (2005). Identification of synaptic plasma membrane proteins co-precipitated with fibrillar beta-amyloid peptide.. J Neurochem.

[31] Bulliard C, Zurbriggen R, Tornare J, Faty M, Dastoor Z (1997). Purification of a dichlorophenol-indophenol oxidoreductase from rat and bovine synaptic membranes: tight complex association of a glyceraldehyde-3-phosphate dehydrogenase isoform, TOAD64, enolase-gamma and aldolase C.. Biochem J.

[32] Ueta H, Nagasawa H, Oyabu-Manabe Y, Toida K, Ishimura K (2004). Localization of enolase in synaptic plasma membrane as an alphagamma heterodimer in rat brain.. Neurosci Res.

[33] Butterfield DA, Lange ML (2009). Multifunctional roles of enolase in Alzheimer's disease brain: beyond altered glucose metabolism.. J Neurochem.

[34] Butterfield DA, Reed T, Newman SF, Sultana R (2007). Roles of amyloid beta-peptide-associated oxidative stress and brain protein modifications in the pathogenesis of Alzheimer's disease and mild cognitive impairment.. Free Radic Biol Med.

[35] Dale RC, Candler PM, Church AJ, Wait R, Pocock JM (2006). Neuronal surface glycolytic enzymes are autoantigen targets in post-streptococcal autoimmune CNS disease.. J Neuroimmunol.

[36] Bigl M, Bruckner MK, Arendt T, Bigl V, Eschrich K (1999). Activities of key glycolytic enzymes in the brains of patients with Alzheimer's disease.. J Neural Transm.

[37] Lovell MA, Xiong S, Markesbery WR, Lynn BC (2005). Quantitative proteomic analysis of mitochondria from primary neuron cultures treated with amyloid beta peptide.. Neurochem Res.

[38] Stamatakis K, Sanchez-Gomez FJ, Perez-Sala D (2006). Identification of novel protein targets for modification by 15-deoxy-delta12,14-prostaglandin J2 in mesangial cells reveals multiple interactions with the cytoskeleton.. J Am Soc Nephrol.

[39] Pelham HR (1984). Hsp70 accelerates the recovery of nucleolar morphology after heat shock.. Embo J.

[40] Whatley SA, Leung T, Hall C, Lim L (1986). The brain 68-kilodalton microtubule-associated protein is a cognate form of the 70-kilodalton mammalian heat-shock protein and is present as a specific isoform in synaptosomal membranes.. J Neurochem.

[41] Schuller E, Gulesserian T, Seidl R, Cairns N, Lube G (2001). Brain t-complex polypeptide 1 (TCP- 1) related to its natural substrate beta1 tubulin is decreased in Alzheimer's disease.. Life Sci.

[42] Baumann MH, Wisniewski T, Levy E, Plant GT, Ghiso J (1996). C-terminal fragments of alpha- and beta-tubulin form amyloid fibrils in vitro and associate with amyloid deposits of familial cerebral amyloid angiopathy, British type.. Biochem Biophys Res Commun.

[43] Islam K, Levy E (1997). Carboxyl-terminal fragments of beta-amyloid precursor protein bind to microtubules and the associated protein tau.. Am J Pathol.

[44] Oyama R, Yamamoto H, Titani K (2000). Glutamine synthetase, hemoglobin alpha-chain, and macrophage migration inhibitory factor binding to amyloid beta-protein: their identification in rat brain by a novel affinity chromatography and in Alzheimer's disease brain by immunoprecipitation.. Biochim Biophys Acta.

[45] Arnaud LT, Myeku N, Figueiredo-Pereira ME (2009). Proteasome-caspase-cathepsin sequence leading to tau pathology induced by prostaglandin J2 in neuronal cells.. J Neurochem.

[46] Alonso A, Zaidi T, Novak M, Grundke-Iqbal I, Iqbal K (2001). Hyperphosphorylation induces self-assembly of tau into tangles of paired helical filaments/straight filaments.. Proc Natl Acad Sci U S A.

[47] Fulga TA, Elson-Schwab I, Khurana V, Steinhilb ML, Spires TL (2007). Abnormal bundling and accumulation of F-actin mediates tau-induced neuronal degeneration in vivo.. Nat Cell Biol.

[48] Morgan TE, Lockerbie RO, Minamide LS, Browning MD, Bamburg JR (1993). Isolation and characterization of a regulated form of actin depolymerizing factor.. J Cell Biol.

[49] Bamburg JR, Bloom GS (2009). Cytoskeletal pathologies of Alzheimer disease.. Cell Motil Cytoskeleton.

[50] Ching GY, Liem RK (1998). Roles of head and tail domains in alpha-internexin's self-assembly and coassembly with the neurofilament triplet proteins.. J Cell Sci.

[51] Kaplan MP, Chin SS, Fliegner KH, Liem RK (1990). Alpha-internexin, a novel neuronal intermediate filament protein, precedes the low molecular weight neurofilament protein (NF-L) in the developing rat brain.. J Neurosci.

[52] Chow SK, Yu D, Macdonald CL, Buibas M, Silva GA (2010). Amyloid-beta directly induces spontaneous calcium transients, delayed intercellular calcium waves, and gliosis in rat cortical astrocytes.. ASN Neuro.

[53] Giri S, Rattan R, Singh AK, Singh I (2004). The 15-deoxy-delta12,14-prostaglandin J2 inhibits the inflammatory response in primary rat astrocytes via down-regulating multiple steps in phosphatidylinositol 3-kinase-Akt-NF-kappaB-p300 pathway independent of peroxisome proliferator-activated receptor gamma.. J Immunol.

