# Correction: Proteomic Identification of Protein Targets for 15-Deoxy-Δ^12,14^-Prostaglandin J_2_ in Neuronal Plasma Membrane

**DOI:** 10.1371/annotation/ce01aaca-d54e-4042-9135-27d755605f4b

**Published:** 2011-04-06

**Authors:** Yasuhiro Yamamoto, Kenkichi Takase, Junji Kishino, Megumi Fujita, Noboru Okamura, Toshiyuki Sakaeda, Masafumi Fujimoto, Tatsurou Yagami

The authors would like to add the following to the funding information: "This study was supported by Himeji Dokkyo University Grant for Special Research Projects"

